# Local Community Composition Drives Avian *Borrelia burgdorferi* Infection and Tick Infestation

**DOI:** 10.3390/vetsci9020055

**Published:** 2022-01-29

**Authors:** Marie Lilly, Wilmer Amaya-Mejia, Lucas Pavan, Ceili Peng, Arielle Crews, Nghia Tran, Ravinder Sehgal, Andrea Swei

**Affiliations:** 1 Department of Biology, San Francisco State University, 1600 Holloway Ave., San Francisco, CA 94132, USA; nghjatran1100@gmail.com (N.T.); sehgal@sfsu.edu (R.S.); 2 Department of Ecology and Evolutionary Biology, University of California, Los Angeles, 612 Charles E. Young Drive East, Los Angeles, CA 90095, USA; amayamejiaws@gmail.com; 3 Department of Biology, Stanford University, 371 Jane Stanford Way, Stanford, CA 94305, USA; lucas.i.pavan@gmail.com; 4 Department of Biology, Massachusetts Institute of Technology, 31 Ames St., Cambridge, MA 02142, USA; ceilipeng@gmail.com; 5 San Mateo County Mosquito and Vector Control District, 1351 Rollins Road, Burlingame, CA 94010, USA; arielle.crews@gmail.com

**Keywords:** disease ecology, community ecology, avian hosts, Lyme disease, *Borrelia burgdorferi*

## Abstract

Globally, zoonotic vector-borne diseases are on the rise and understanding their complex transmission cycles is pertinent to mitigating disease risk. In North America, Lyme disease is the most commonly reported vector-borne disease and is caused by transmission of *Borrelia burgdorferi* sensu lato (s.l.) from *Ixodes* spp. ticks to a diverse group of vertebrate hosts. Small mammal reservoir hosts are primarily responsible for maintenance of *B. burgdorferi* s.l. across the United States. Nevertheless, birds can also be parasitized by ticks and are capable of infection with *B. burgdorferi* s.l. but their role in *B. burgdorferi* s.l. transmission dynamics is understudied. Birds could be important in both the maintenance and spread of *B. burgdorferi* s.l. and ticks because of their high mobility and shared habitat with important mammalian reservoir hosts. This study aims to better understand the role of avian hosts in tick-borne zoonotic disease transmission cycles in the western United States. We surveyed birds, mammals, and ticks at nine sites in northern California for *B. burgdorferi* s.l. infection and collected data on other metrics of host community composition such as abundance and diversity of birds, small mammals, lizards, predators, and ticks. We found 22.8% of birds infected with *B. burgdorferi* s.l. and that the likelihood of avian *B. burgdorferi* s.l. infection was significantly associated with local host community composition and pathogen prevalence in California. Additionally, we found an average tick burden of 0.22 ticks per bird across all species. Predator and lizard abundances were significant predictors of avian tick infestation. These results indicate that birds are relevant hosts in the local *B. burgdorferi* s.l. transmission cycle in the western United States and quantifying their role in the spread and maintenance of Lyme disease requires further research.

## 1. Introduction

Globally, zoonotic vector-borne diseases have emerged over the past several decades and constitute a major public health challenge [1,2,3,4]. In the United States, Lyme disease is the most commonly reported vector-borne disease to the Center for Disease Control and Prevention (CDC) and the number of human Lyme disease cases has doubled over the last 16 years, currently estimated to exceed 300,000 cases [5]. Lyme disease is maintained by the transmission of the spirochete bacteria *Borrelia burgdorferi* sensu stricto (s.s.) between Ixodes spp. ticks and their vertebrate hosts [6,7,8]. The *B. burgdorferi* sensu lato species complex consists of 18 related genospecies, some of which are zoonotic and others are not. In the United States, *B. burgdorferi* s.s. and a newly discovered, *B. mayonii*, are the only genospecies known to cause Lyme disease [9,10] but other genospecies, such as *B. bissettiae* have been associated with human disease [11,12].

In the western United States, the primary vector of Lyme disease is the western black-legged tick, *Ixodes pacificus* [8], a generalist tick that can feed on over 100 species of mammals, birds, and reptiles [13,14,15]. Some of these so-called reservoir hosts can become infected with *B. burgdorferi* s.l., amplify the bacteria, and transmit it to uninfected feeding ticks—thus maintaining *B. burgdorferi* s.l. in the community [16]. A few key mammalian host species identified as *B. burgdorferi* s.l. reservoirs in the western United States include the western gray squirrel (*Sciurus griseus*), dusky-footed woodrat (*Neotoma fuscipes*), and deer mice (*Peromyscus* spp.) [14,16,17,18,19,20]. Birds are capable of both sustaining *B. burgdorferi* s.l. infections and transmitting infection to ticks, but the extent of their role in *B. burgdorferi* s.l. transmission dynamics is not well understood [21,22,23,24,25]. Birds are important sylvatic reservoirs of *B. burgdorferi* s.l. in parts of Europe, but few studies in North America have quantified the role of birds as tick hosts and pathogen reservoirs [26,27,28,29]. Experimental studies have shown that birds can effectively transmit *B. burgdorferi* s.l. infection to ticks and have been implicated as important carriers of *Ixodes* spp. ticks between different geographic regions [22,30,31]. Thus, birds may be important in both maintenance and spread of *B. burgdorferi* s.l. because of their high mobility and shared habitat with important mammalian reservoir hosts [15,21,22].

*Ixodes* spp. have three post egg life stages, the larva, nymph, and adult stages. Because *Borrelia burgdorferi* s.l. is not transovarially (vertically) transmitted from adult tick to larvae, the identity and infection status of the larval bloodmeal is the primary determinant of the infection status of nymphal ticks [32]. The nymphal tick poses the greatest threat to human disease transmission because of its small size and the ease with which it might be overlooked, even after a thorough tick check [33,34,35]. Thus, *B. burgdorferi* s.l. infection status of nymphal ticks is commonly used as an entomological metric for *B. burgdorferi* s.l. disease risk. Birds predominantly host larval and nymphal ticks, but their tick burden and ability to transmit *B. burgdorferi* s.l. to feeding ticks varies greatly by species and geographic region [24,31,36,37].

This study aims to resolve unanswered questions regarding ecological drivers of avian *B. burgdorferi* s.l. infection prevalence and avian tick burden to better understand the role of avian hosts in tick-borne zoonotic disease transmission cycles. We sought to assess how community composition across trophic scales—from tick vector to small mammal to predator communities—affect avian tick burdens and infection prevalence with *B. burgdorferi* s.l. We also ask if certain avian traits are important for predicting tick burden and the likelihood of *B. burgdorferi* s.l. infection. This study adds to the growing body of knowledge regarding the understudied role of avian hosts in *B. burgdorferi* s.l. transmission dynamics.

## 2. Materials and Methods

### 2.1. Site Selection

**Figure 1:**
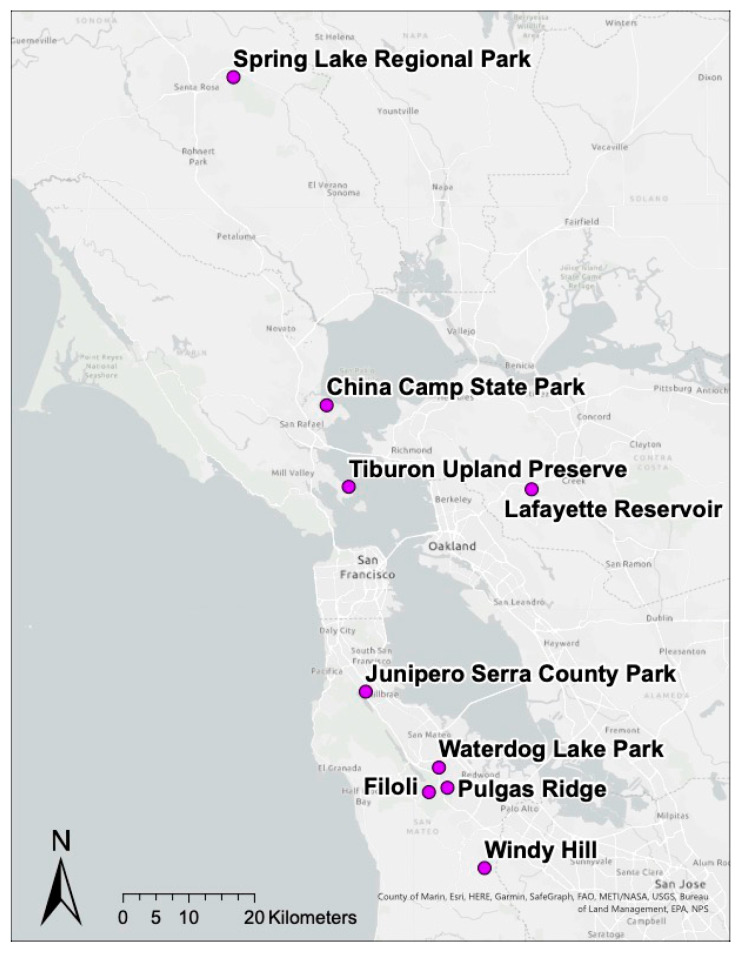
Map of nine sites across Northern California where field plots were established for Lyme disease surveillance and bird, mammalian, tick, and reptilian communities were sampled.

Birds were surveyed on field plots from nine oak woodland habitat sites in northern California that were selected to span a patch size gradient as described by Lawrence et al. (2018) [38] and capture a range of *I. pacificus* host community diversity and composition. Five sites were located in San Mateo County, CA, USA (Filoli Estates (37.46946, −122.31639), Junipero Serra County Park (37.60792, −121.42417), Water Dog Lake Park (37.50311, −122.29889), Windy Hill Open Space Preserve (37.86514, −122.2801), Pulgas Ridge Preserve (37.474749, −122.285120)); two sites were located in Marin County, CA, USA (China Camp State Park (38.00131, −122.48889), Tiburon Uplands Nature Preserve (37.88931, −121.45083)); one site was located in Contra Costa County, CA, USA (Lafayette Reservoir Nature Area (37.88394, −122.13472)); and one site was located in Sonoma County, CA, USA (Spring Lake Regional Park (38.45225, −122.64833)) (Figure 1). Birds were sampled from April to August of 2019 while the tick, mammalian, and reptilian communities were sampled from April to May in 2019 during peak questing activity of *I. pacificus* larvae and nymphs [8,39].

### 2.2. Host Surveillance: Birds, Small Mammals, Lizards, and Medium to Large Mammals

Bird communities were sampled using standard mist-netting techniques (Stanford University IACUC protocol #33733) [40]. Twelve mist-nets, measuring 12 m long by 3 m high, were set up along trails or open spaces in a perpendicular array near the forest edge. Mist netting occurred from 7 am to noon for two consecutive days at each site and were checked at 30 min intervals. Birds were extracted from the net, identified to species [41,42], banded, sexed, and aged [43]. Morphometrics were recorded for weight, fat score, and bill, tarsus, wing, and tail length. Approximately 5–20 μL of blood for molecular analyses was collected by brachial venipuncture of each captured bird and stored in lysis buffer (10 mM Tris-HCl pH 8.0, 100 mm ethylenediaminetetraacetic acid, 2% sodium dodecyl sulfate) [43,44]. Although *B. burgdorferi* s.l. is known to colonize mammalian tissue more readily than blood, spirochetes have been found in avian blood analyses, so this standard avian sampling technique was chosen over a more invasive skin biopsy approach [21,45].

Small mammals were trapped at each site on a half-hectare grid using a 7 × 7 trapping array (San Francisco State University IACUC protocol #AU19-01R2). Two Sherman traps were set at each trapping station facing opposite directions from each other with 11.8 m spacing between each of the 49 sampling stations. Each grid and trapping array were standardized as described by Lawrence et al. (2018) [38] and Salomon et al. (2021) [46]. Small mammals were trapped for three consecutive days at each site. Each animal caught was identified to species in the field and confirmed by molecular analysis of the *cytochrome b* gene. They were visually sexed, weighed, and uniquely tagged. A 2 mm circular tissue biopsy was taken from the outer pinna of each ear and immediately stored in 70% ethanol.

For each site, western fence lizards (*Sceloporus occidentalis*) were visually surveyed within the 0.5 ha sampling plot along seven evenly spaced transect lines with 11.8 m between each line. Lizards were sprayed using Idico (Forestry Suppliers, Jackson, MS, USA) tree-marking guns on their dorsum with a diluted latex paint mixture as described by Swei et al., 2011 [47]. Lizard surveys took place over three consecutive days with a different paint color used on each day to determine individual lizard’s encounter history.

Motion sensor wildlife camera traps (Bushnell models #119736, #119836, #119836C, Bushnell, KS, USA) were set in open spaces and along game trails at each site to capture the relative abundance and movement of predators and other medium to large mammals. Two camera traps were set for a sampling period of 40 days at each site on the same tree facing opposite directions as described by Lawrence et al. (2018) [38]. Animals captured by the camera traps were identified to species by visualizations of the photographs. 

Vertebrate species abundance and diversity were calculated at each site. Relative abundance of birds was estimated as the minimum number alive of each species caught in the mist nets at all nine sites [40,48]. Small mammal and western fence lizard abundances were estimated from the mark-recapture trapping data using the R package *Rcapture* [49]. The relative abundance of predators was calculated by counting the number of unique individuals of each species per photograph per day across the 40-day trapping period [39,46]. Avian, rodent, and predator Shannon diversity were calculated in R using the *vegan* package, and richness was calculated as the number of unique species captured [50].

### 2.3. Vecotor Surveillance: Bird-Attached and Questing Ticks

Birds sampled were checked for ticks around the head and all attached ticks were removed and stored in 70% ethanol for later identification to species and life stage by microscopy using taxonomic keys [51,52,53].

Questing ticks were collected by standard dragging techniques using a 1 m^2^ white cotton cloth along seven linear transects within the 0.5 ha sampling plot for a total of 495 m^2^ sampled at each site [54]. All ticks were stored in 70% ethanol and identified to species and life stage by microscopy using taxonomic keys [52,53]. Questing *I. pacificus* nymphal tick abundance was calculated as the total number of *I. pacificus* nymphal ticks per drag at each site.

### 2.4. Pathogen Surveillance: Borrelia burgdorferi Sensu Lato Testing

DNA was extracted from avian blood samples, small mammal ear tissue biopsies, and questing nymphal ticks for molecular analyses using either Qiagen DNeasy Blood and Tissue Kit (Qiagen, Valencia, CA, USA) or Promega Wizard Genomic DNA Purification Kit (Promega, Madison, WI, USA). Extracted DNA from each sample was screened for *B. burgdorferi* s.l. with a nested PCR protocol targeting the 5S-23S rRNA intergenic spacer region [55] and then visualized by gel electrophoresis. Positive samples were then identified to genospecies by sequencing (GenBank MZ852103-MZ852212). Four samples did not have enough PCR product for sequencing and were counted as *B. burgdorferi* s.l. positive based on the strength of the amplicon banding during gel electrophoresis but not identified to genospecies.

### 2.5. Statistical Analyses: Probability of Avian Tick Infestation and Borrelia burgdorferi Sensu Lato Infection

Statistical analyses were performed to assess the avian traits and overall community composition metrics important in driving avian tick burden and the likelihood of *B. burgdorferi* s.l. infection. Only bird species with a sample size of ≥5 individuals tested and with ≥1 infected individual were included in subsequent analyses. To address what host traits influence avian tick burden, a zero-inflated negative binomial generalized linear mixed-effect model (GLMM) was used to analyze the effect of bird species, sex, mass, foraging substrate, nesting substrate, and *B. burgdorferi* s.l. infection status on avian tick burden. Each avian species was categorized by preferred foraging substrate and nesting type based on prior studies [15,41,56] with site included as a random effect. Models were implemented in the R package *glmmTMB* [57]. Model family was chosen based on the data distribution and the best models were chosen by comparing Akaike Information Criterion (AIC) scores. A two-sample t-test assuming unequal variance was also performed in excel to calculate the difference in tick burden between ground-dwelling birds and birds that forage and nest above ground.

Vertebrate community composition impact on avian tick burden was analyzed with a zero-inflated negative binomial GLMM with avian tick burden as the response variable and vertebrate species abundance, richness, and diversity as fixed effects. Models were again created using the R package *glmmTMB* and the model family was chosen based on the data distribution. The best models were chosen by comparing AIC scores.

To address what avian traits and host community parameters influence the probability of avian *B. burgdorferi* s.l. infection, binomial GLMM analyses were used with avian *B. burgdorferi* s.l. infection status set as a binary response variable. We constructed three separate GLMM models to address how avian *B. burgdorferi* s.l. infection is predicted by (1) natural history and avian demographic variables, (2) vertebrate community structure, specifically richness, and (3) pathogen prevalence in hosts and ticks. Separate models were constructed to not overparameterize the models and because host community richness and host prevalence variables were collinear. In addition, all metrics of tick infection prevalence, density of nymphs (DON), nymphal infection prevalence (NIP), and density of infected nymphs (DIN) were correlated for data collected in 2019, the year that bird data were collected, and for 2018, the previous year. Because of the life cycle of the tick, host infection prevalence for long lived species can sometimes be better predicted by lagged data, i.e., nymph infection prevalence in the previous year [58]. For all models, fixed effects for each model were checked for collinearity and site was included as a random effect. We first examined the relationship between avian infection prevalence and host life history traits in what we call model 1. This model included bird species, sex, mass, foraging and nesting substrate category, and tick burden as fixed effects. Next, we examined the role of vertebrate community composition in model 2 which included avian richness, rodent richness, and predator richness as fixed effects. Lastly, we explored the influence of reservoir host infection prevalence and the potential for a lagged effect in model 3 which included *N. fuscipes* infection prevalence with *B. burgdorferi* s.l. and the nymphal infection prevalence from both the current year (2019) and the previous year (2018). Model comparison was conducted using AIC scores to determine the most parsimonious model. Host infection analysis focused on *N. fuscipes* infection prevalence only because *Peromyscus* infection prevalence was highly correlated with *N. fuscipes* infection prevalence and because *N. fuscipes* is a more important contributor to local *B. burgdorferi* s.l. transmission [17].

## 3. Results

### 3.1. Host Community Composition: Birds, Small Mammals, and Medium to Large Mammals

Across all nine sites, 160 individual birds belonging to 23 different species were caught and processed. Statistical analysis of avian tick burden focused on 118 individual birds from six bird species that included individuals infected with *B. burgdorferi* s.l. and had a sample size ≥5 individuals across all sites: the oak titmouse (*Baeolophus inornatus*), spotted towhee (*Pipilo maculatus*), pacific slope flycatcher (*Empidonax difficilis*), dark-eyed junco (*Junco hyemalis*), Bewick’s wren (*Thryomanes bewickii*), and lesser goldfinch (*Spinus psaltria*) (Table 1). Of these six species examined, the pacific slope flycatcher is a migratory species through the western United States while the remaining five are non-migratory resident birds in the region [41].

Statistical analyses of avian *B. burgdorferi* s.l. infection focused on these same six bird species but excluded 17 individuals (101 total birds) that were checked for ticks but unable to have blood drawn.

Across all sites, five different small mammal species were caught; the dusky-footed woodrat (*Neotoma fuscipes*), deer mouse (*Peromyscus maniculatus*), California deer mouse (*Peromyscus californicus*), pinyon mouse (*Peromyscus truei*), and western harvest mouse (*Reithrodontomys megalotis*). Total abundance of *N. fuscipes* was estimated to be 51 individuals across all sites (Tables S1 and S2). Western fence lizards (*Sceloporus occidentalis*) were observed at six of the nine sites and overall abundance was estimated to be 299 individuals (Table S1). Our wildlife cameras detected six terrestrial mammal species known to prey upon small birds that were captured on our camera traps: the bobcat (*Lynx rufus*), gray fox (*Urocyon cinereoargenteus*), coyote (*Canis latrans*), skunk (*Mephitis mephitis*), raccoon (*Procyon lotor*), and opossum (*Didelphis virginiana*) [59]. 

### 3.2. Vector Suveillance: Bird-Attached and Questing Ticks

A total of 26 bird-attached *I. pacificus* ticks were removed from 13 individual birds, with 11 larvae and 15 nymphal ticks from the bird species included in analyses for an average tick burden of 0.22 ticks per bird (Table 1). Three additional *Haemaphysalis* ticks (Two *H. leporispalustris* and one *H. chordeilis*) were also found attached to the birds but were not included in our analyses. A total of 338 questing nymphal *I. pacifus* ticks were collected and tested for *B. burgdorferi* s.l. (Table S2).

### 3.3. Pathogen Surveillance: Borrelia burgdorferi Sensu Lato Prevalence

The overall avian *B. burgdorferi* s.l. infection prevalence across all sites and species was 22.8%, (N = 23/101). One dark-eyed Junco (*Junco hyemalis*) was infected with *B. bissettiae* while 18 birds were infected with *B. burgdorferi* s.s. There were no birds coinfected with multiple Borrelia species. Four birds included in *B. burgdorferi* s.l. analysis tested positive for *B. burgdorferi* s.l. but did not yield informative sequence results. The overall *B. burgdorferi* s.l. infection prevalence of *N. fuscipes* was 29.5% while the overall nymphal *I. pacificus* tick infection prevalence was 15% across all nine sites (Table S2).

### 3.4. Statistical Analysis: Probability of Avian Tick Burden

Avian tick burden was not significantly correlated with foraging or nesting substrate [15,60] (Figure S1). We did not find an association between tick burden and avian-associated demographic or morphometric traits either such as weight, sex, or species.

**Figure 2:**
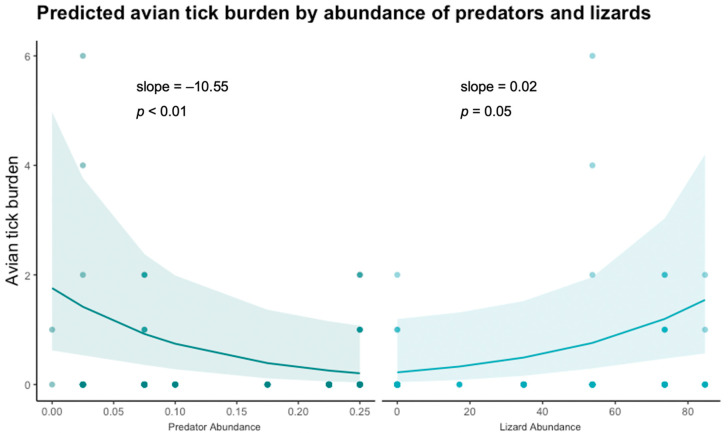
Zero-inflated Poisson distributed generalized linear model result of relative predator abundance and *S. occidentalis* lizard abundance as predictors of avian tick burden with site as a random effect. Raw data jittered over model prediction.

Of the host community characteristics examined, relative predator abundance and *S. occidentalis* abundance were significant predictors of intensity of avian tick burden (predator abundance estimate = −10.55, *p* < 0.001; lizard abundance estimate = 0.02, *p* = 0.05) (Figure 2, Table 2). There was no significant pattern found between avian tick burden and abundance of small mammals or abundance of birds detected in our study (Table 2). Other elements of host community composition including Shannon diversity and richness of: predators, small mammals, birds, and lizards, were not significant predictors of avian tick burden.

**Figure 3:**
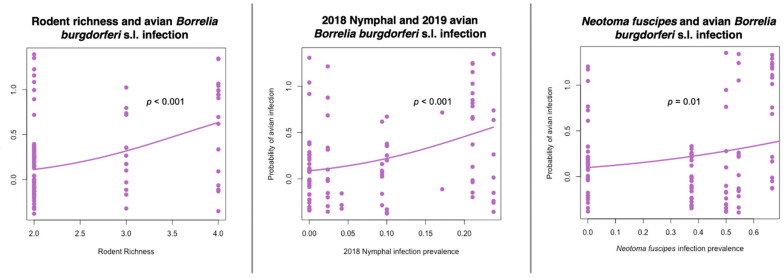
Rodent community composition and tick infection prevalence significantly predicts the probability of avian *Borrelia burgdorferi* sensu lato infection. Raw data jittered over model predictions.

The model results of GLMM analyses did not find a significant relationship between host natural history or demographic variables on the probability of avian infection with *B. burgdorferi* (model 1). In model 2 we found a significant and positive correlation between rodent species richness and the probability of *B. burgdorferi* s.l. infection in birds, but bird and predator species richness were not significant predictors (Table 3). Reservoir host infection analysis (model 3) focused on *N. fuscipes* infection prevalence as well as nymphal *B. burgdorferi* s.l. infection prevalence (NIP) and showed that *N. fuscipes* infection prevalence in 2019 and NIP in 2018 were the most significant and parsimonious predictors of probability of avian *B. burgdorferi* s.l. infection in 2019 (Figure 3, Table 3).

## 4. Discussion

The etiological agent of Lyme disease is maintained by a complex enzootic cycle that requires a tick vector and competent reservoir hosts to persist [6]. While many studies have focused on the role of rodents in Lyme disease transmission because of their importance and competence as reservoir hosts, birds are relatively understudied but may be important in the sylvatic cycle of Lyme disease. This study sought to determine the host and community composition traits that affect the role of birds as both tick hosts and *B. burgdorferi* s.l. pathogen reservoirs. Not only does our study assess the tick infestation and *B. burgdorferi* s.l. infection prevalence of wild birds, but we examine their role in the context of a local enzootic cycle. Our analyses found that local terrestrial predator and western fence lizard abundance are predictive of intensity of avian tick infestation. Despite the high vagility of birds, we found that avian *B. burgdorferi* s.l. infection was highly predicted by rodent richness and site-specific rodent and tick infection prevalence. These results show that bird infection prevalence is highly concordant with local mam-mal and tick infection, indicating that they are likely acquiring infection locally. By showing that avian tick burden and *B. burgdorferi* s.l. infection status is associated with elements of local community composition, our study confirms that birds, especially the resident bird species examined in this study, are potentially important hosts for maintaining local *Borrelia burgdorferi* sensu lato transmission dynamics in the western United States.

Tick burden is an important determinant of whether a host is a competent *B. burgdorferi* s.l. reservoir. The tick burdens of the avian species in our study had a low average (0.22) but wide range of 0–6 ticks per bird, consistent with other studies of avian tick burdens across North America [15,60,61]. However, due to logistical constraints, the timing of avian sampling extended three months beyond the peak nymphal and larval questing period, so our burden averages are likely an underrepresentation of avian tick burden. Contrary to previous studies, intensity of tick infestation was not significantly associated with avian morphometrics nor life history traits [15,19,36,60,61,62]. However, it is possible that limited sample size or our study design which only included six distinct bird species in analysis may not have provided a robust enough species sampling to uncover patterns related to species specific life history and morphometric traits.

We found that predator abundance was a significant, negative driver of avian tick burden with tick infestation decreasing as predator abundance increased. These results are consistent with recent findings that small mammal tick burdens are negatively impacted by predator composition [46]. Our results suggest that although predators can regulate tick populations by limiting host availability, this was not the case for our study system because we did not find a negative effect of predator abundance on host populations [46]. Another possible mechanism to explain this pattern is supported by the ‘ecology of fear’ hypothesis [63]. In the presence of a high density of predators, birds might spend less time on the ground foraging or resting where they are more likely to encounter ticks [15,19,64,65,66,67,68,69]. This hypothesis is supported by a large body of literature indicating that increased levels of predation cause prey to spend more time engaged in predator avoidance and vigilance behaviors and less time on the ground foraging [70,71,72,73,74]. By reducing their nesting density and foraging behavior in the presence of high terrestrial predator activity [49], ground-dwelling birds would also reduce encounters with ticks. Behavioral observation studies would be useful to shed light on this idea.

In addition to local predator abundance, we found that western fence lizard (*S. occidentalis*) abundance was a positive predictor of avian tick burden. *Sceloporus occidentalis* are the primary *I. pacificus* tick host in the western United States and lizard presence has been experimentally shown to influence local tick population dynamics [19,75]. Although abundance of questing of *I. pacificus* nymphs alone was not a significant driver of avian tick burden, it is possible that the lizard population drives the total tick population (including both questing ticks and those already attached to available hosts) and thus lizard abundance may lead to an increase in total ticks present at each site.

The high vagility of birds suggests that they may be important for long distance dispersal of ticks or pathogens between remote geographic locations, however our study indicates that birds are more likely acquiring infections locally based on their significant association with site-specific rodent richness, *N. fuscipes* infection prevalence, and nymphal tick infection prevalence (Table 3) [21,31,60,62,76,77]. This is consistent with the idea that the likelihood of a bird encountering an infected tick and becoming infected would be higher where there is greater transmission of *B. burgdorferi* s.l. at the community level. *Neotoma fuscipes* are a primary reservoir host for *B. burgdorferi* s.l. and thus their infection prevalence should reflect rates of overall risk of *B. burgdorferi* s.l. Avian *B. burgdorferi* s.l. infection prevalence was significantly associated with NIP of the previous year, but not NIP of the concurrent avian sampling year. This could indicate that birds, with lifespans of 2 to 11 years, may be acquiring infection in the previous season [41]. Alternatively, these results could reflect variability in detection of infection between the tick population and avian blood samples.

Overall avian infection prevalence (22.8%) is comparable to other known reservoir host species such as *N. fuscipes* (29.5%). Due to the limitations of detecting *B. burgdorferi* s.l. in blood compared to tissue [28], these infection rates may be an underestimate of avian infection prevalence. The ability of birds to serve as competent reservoirs in local transmission dynamics relies on the ability of these infected birds to transmit *B. burgdorferi* s.l. to a naïve feeding tick, a component of transmission that we did not measure in this study. An experimental study conducted by Richter et al. (2000) [30] found that laboratory infected American robins (*Turdus migratorius*) were comparable to rodents at acquiring and transmitting *B. burgdorferi* s.l. infection to ticks, suggesting that birds could be reservoir hosts for *B. burgdorferi* s.l., however, the experiment found that the infection status of robins waned more rapidly than that of rodents [30]. Future studies should further investigate the reservoir capacity of avian hosts through xenodiagnostic experiments of transmission efficiency of *B. burgdorferi* s.l. between birds and ticks.

While our study found relatively high rates of *B. burgdorferi* s.l. infection among avian hosts, we did not assess the impact of *B. burgdorferi* s.l. on avian fitness. Among birds, there is high interspecific variability in tolerance versus resistance to different parasites [78,79,80,81]. Although laboratory experiments artificially infecting birds with *B. burgdorferi* s.l. have shown no clinical symptoms of *Borrelia* infection nor detectable impact on avian health, the long-term impact of *B. burgdorferi* s.l. infection on wild avian host fitness remains to be determined [21,28]. The long-term avian health impact of *B. burgdorferi* s.l. infection could have important implications for their overall reservoir competency [30]. Birds typically have a much longer lifespan than small mammals, with the bird species we sampled living up to maximums of 7–11 years compared to 1–3 years for our rodent reservoir hosts such as *Peromyscus* mice and woodrats [41,82]. This difference in lifespan, if combined with persistent infection, could result in birds playing a more important role in pathogen maintenance over the course of their life. If one exposure event to an infected tick results in a persistent infection over the life of the bird, birds could be especially important for carrying the pathogen between years and maintaining rare *B. burgdorferi* s.l. genotypes in the population [25,32,83,84,85,86]. Our study found both *B. burgdorferi* and *Borrelia bissettiae* in avian hosts, confirming a finding by Newman et al. (2015) [15]. Additional studies on the persistence of other genospecies of *B. burgdorferi* s.l. in birds would greatly improve our understanding of the potential life-long contribution of birds to pathogen transmission and diversity.

While our study focused mostly on resident songbird communities that are not likely to move more than 15–95 km, one species included in our analyses, the pacific slope flycatchers, are part of the over 65 million land birds that migrate through California each year and could transport ticks and *B. burgdorferi* s.l. during their journey and underscore the importance of studying both resident and migratory birds [60,87,88,89,90]. Ogden et al. (2008) [31] found that migratory birds transport 50–175 million ticks across Canada each spring, with a *B. burgdorferi* s.l. infection prevalence of 15.8% in attached nymphs. While it is unclear if ticks are able to establish after relocation via avian hosts, there is clear potential for birds to increase existing tick populations and introduce novel genetic variants of *B. burgdorferi* s.l. [90]. Additionally, migratory birds often interact with resident bird populations and rely on them for finding suitable foraging and nesting sites [88,91]. The high prevalence of *B. burgdorferi* s.l. detected in bird species in our study could lead to an increased likelihood of pathogen spread to migratory birds through shared habitat and spread of pathogen infected attached ticks to new regions [28,31].

## 5. Conclusions

Our study examines how complex community interactions drive avian *B. burgdorferi* s.l. infection prevalence and tick parasitism among resident bird populations in the western United States. Many of the bird species that were examined in this study are resident species with limited dispersal behavior and our analyses indicate that these birds are likely acquiring infection locally. Our findings that rodent richness is driving avian *B. burgdorferi* s.l. infection status and that predator and lizard abundances are driving avian tick burden highlight the importance of considering full community dynamics when evaluating birds, or any other host, as a potential *B. burgdorferi* s.l. reservoir. Future studies should focus on the reservoir competency and ability of key bird species to infect ticks in order to further resolve the role of birds in local *B. burgdorferi* s.l. transmission dynamics and their potential for maintaining and spreading *B. burgdorferi* s.l. to new environments.

## Figures and Tables

**Table 1:** Avian host community sampling distribution, *Borrelia burgdorferi* s.l. infection prevalence, foraging and nesting substrate by bird species included in analysis.

Species	N Tested for *B. burgdorferi* s.l.	N *B. burgdorferi* s.l. Infected (%)	Average Tick Burden	Foraging Substrate	Nesting Substrate
Bewick’s Wren	16 (11)	2 (18)	0	Aboveground	Aboveground
Dark-eyed Junco	67 (65)	14 (21.5)	0.28	Ground	Ground
Lesser Goldfinch	6 (5)	2 (40)	0	Aboveground	Aboveground
Oak Titmouse	11 (7)	1 (14)	0.55	Aboveground	Aboveground
Pacific Slope Flycatcher	6 (5)	2 (40)	0	Aboveground	Aboveground
Spotted Towhee	12 (8)	2 (25)	0.08	Ground	Ground
Totals	118 (101)	23 (22.8)	0.22		

**Table 2:** Zero-inflated Poisson generalized linear model results of host community characteristics as predictors of avian tick burden with site as a random variable.

Response Variable	Model Component	Estimate	Standard Error	Z-Value	*p*-Value
Avian tick burden	(Intercept)	−0.15	0.99	−0.15	0.88
	**Predator abundance**	**−10.55**	**3.28**	**−3.22**	0.001 **
	Bird abundance	0.04	0.03	1.11	0.26
	*N. fuscipes* abundance	−0.06	0.06	−1.05	0.29
	***S. occidentalis* abundance**	**0.02**	**0.01**	**0.77**	0.05 *

* and ** denote significance level.

**Table 3:** Binomial model results of natural history and host demographic variables, and host community characteristics as predictors of avian *B. burgdorferi* s.l. infection status with site as a random variable.

Model Number	Response Variable	Model Component:	Estimate	Standard Error	Z-Value	*p*-Value
Model 1	Avian *B. burgdorferi* s.l. infection	(Intercept)	−1.81	0.94	−1.91	0.06
		Bird species:				
		*J. hyemalis*	0.16	1	0.16	0.87
		*S. psaltria*	−0.73	1.48	−0.49	0.62
		*B. inornatus*	−1.62	1.59	−1.02	0.3
		*E. difficilis*	1.23	1.58	0.78	0.43
		*P. maculatus*	0.95	1.45	0.66	0.51
		Sex:				
		Male	0.26	0.82	0.32	0.75
		Unknown	0.16	0.7	0.23	0.82
		Mass	0.01	0.02	0.36	0.72
		Foraging and nesting substrate:				
		Aboveground	−0.4	0.67	−0.6	0.54
		Tick burden	0.32	0.59	0.55	0.58
Model 2	Avian *B. burgdorferi* s.l. infection	**(Intercept)**	**−4.28**	**1.3**	**−3.3**	<0.001 ***
		Avian richness	0.009	0.1	0.1	0.92
		**Rodent richness**	**1.3**	**0.33**	**3.91**	<0.001 ***
		Predator richness	−0.26	0.39	−0.65	0.52
Model 3	Avian *B. burgdorferi* s.l. infection	**(Intercept)**	**−3.25**	**0.59**	**−5.47**	<0.001 ***
		**Nymphal infection prevalence 2018**	**11.13**	**2.79**	**3.95**	<0.001 ***
		***N. fuscipes*** ***B. burgdorferi*** **s.l. infection prevalence 2019**	**2.45**	**1.02**	**2.38**	0.016 *

* and *** denote significance level.

## Data Availability

The data presented in this study are openly available in GenBank (GenBank MZ852103-MZ852212) or contained within the article.

## Supplementary Materials

The following supporting information can be downloaded at: https://www.mdpi.com/article/10.3390/vetsci9020055/s1, Table S1: Community richness and abundance estimates included in analyses across all sites, Table S2: Tick and reservoir host community characteristics included in analysis, Figure S1: Average avian tick burden does not differ by level of ground activity.
