# Supplementary material for: Local Community Composition Drives Avian Borrelia burgdorferi Infection and Tick Infestation
Source: Vet Sci. 2022 Jan 29;9(2):55. doi: 10.3390/vetsci9020055 (PMC8875765; doi:10.3390/vetsci9020055)
Supplement: Supplementary file 1 [file vetsci-09-00055-s001.zip › vetsci-1409203-supplementary.pdf]

Supplemental Materials

Table S1. Community richness and abundance estimates included in analyses across all sites. Avian abundance calculated as minimum number alive and predator abundance calculated as relative abundance by site.

Site	Avian richness	Rodent richness	Predator richness	Avian abundance	*N. fuscipes* abundance	Predator abundance	*S. occidentalis* abundance
China Camp State Park	8	3	1	18	11.5	0.075	84.7
Filoli Estates	2	3	0	2	2	0	0
Junipero Serra County Park	9	2	2	43	10	0.225	0
Lafayette Reservoir Nature Area	9	2	2	14	1	0.1	34.8
Pulgas Ridge Preserve	11	2	1	23	4	0.025	53.7
Spring Lake Regional Park	2	2	2	8	1	0.175	34.8
Tiburon Uplands Nature Preserve	6	2	3	18	15.1	0.075	0
Water Dog Lake Park	2	3	3	3	3	0.25	17.1
Windy Hill Open Space Preserve	11	4	2	31	3	0.25	73.6

Table S2. Tick and reservoir host community characteristics included in analysis. Questing nymphal *Ixodes pacificus* tick surveillance, *Neotoma fuscipes* population surveillance, and bird population surveillance for *Borrelia burgdorferi* s.l infection across all sites.

Site	Questing nymphs tested (N) 2018	Density of infected nymphs 2018	Nymphal infection prevalence 2018	*N. fuscipes* tested (N) 2019	*N. fuscipes* infected (N) 2019	*N. fuscipes* infection prevalence 2019	Birds tested (N) 2019	Birds infected (N) 2019	Bird infection prevalence 2019
China Camp State Park	101	24	23.76%	11	0	0.00%	16	3	18.75%
Filoli Estates	35	5	14.71%	2	0	0.00%	2	1	50.00%
Junipero Serra County Park	1	0	0.00%	8	3	37.50%	36	0	0.00%
Lafayette Reservoir Nature Area	10	1	10.00%	1	0	0.00%	11	1	9.09%
Pulgas Ridge Preserve	NA	NA	NA	4	2	50.00%	19	5	26.32%
Spring Lake Regional Park	64	6	9.38%	1	0	0.00%	8	1	12.50%
Tiburon Uplands Nature Preserve	46	1	2.17%	11	6	54.55%	13	2	15.38%
Water Dog Lake Park	24	1	4.17%	3	0	0.00%	3	0	0.00%
Windy Hill Open Space Preserve	57	12	21.05%	3	2	66.67%	22	12	54.55%

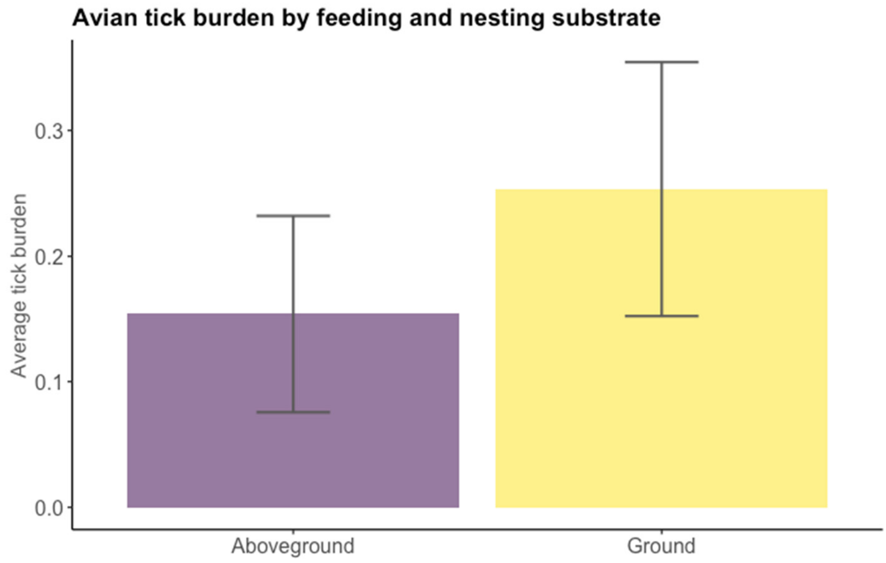

Figure S1. Average avian tick burden does not differ by level of ground activity.
